# Association of nutrition knowledge, practice, supplement use, and nutrient intake with strength performance among Taekwondo players in Nepal

**DOI:** 10.3389/fnut.2022.1004288

**Published:** 2022-11-14

**Authors:** Dev Ram Sunuwar, Devendra Raj Singh, Man Prasad Bohara, Vintuna Shrestha, Kshitij Karki, Pranil Man Singh Pradhan

**Affiliations:** ^1^Department of Nutrition and Dietetics, Armed Police Force Hospital, Kathmandu, Nepal; ^2^School of Human and Health Sciences, University of Huddersfield, Huddersfield, United Kingdom; ^3^Department of Public Health, Asian College for Advance Studies, Purbanchal University, Lalitpur, Nepal; ^4^Department of Nursing, Dhaulagiri Prabhidhik Shikshya Pratisthan, Council for Technical Education and Vocational Training, Baglung, Nepal; ^5^School of Environment Resources and Development, Asian Institute of Technology, Rangsit, Thailand; ^6^Department of Community Medicine, Institute of Medicine, Tribhuvan University, Kathmandu, Nepal; ^7^Nepalese Society of Community Medicine, Kathmandu, Nepal; ^8^Department of Global Health and Population, Harvard T.H. Chan School of Public Health, Boston, MA, United States

**Keywords:** nutritional knowledge, practice, supplement use, nutrient intake, strength performance, Taekwondo player, Nepal

## Abstract

**Background:**

Optimum dietary intake and adequate nutritional knowledge have been recognized as the key factors that play a critical role in improving the athlete's health and nutrition status. This study aimed to measure the association of nutritional knowledge, practice, supplement use, and nutrient intake with strength performance among Nepalese Taekwondo players.

**Methods:**

Between August 2019 and January 2020, a cross-sectional study was conducted among 293 Taekwondo players in Kathmandu Metropolitan City (mean age, 18 years; 63.1% male, 36.9% female). Face-to-face interviews were conducted using semi-structured questionnaires. Anthropometric measures, nutritional intake, nutrition knowledge, and practice were all recorded. The handgrip strength was measured using a handgrip dynamometer as a proxy for strength performance. Univariate and bivariate analyses were used to find out the association between predictor and outcome variables.

**Results:**

More than half of the participants had poor nutrition knowledge [54.3% (159/293)], and poor nutrition practice [55.3% (162/293)] scores. Daily mean energy, carbohydrate, protein and fat intake were 48.0 kcal.kg^−1^.d^−1^, 8.6, 1.6, and 1.5 g.kg^−1^.d^−1^, respectively among Taekwondo players. Daily total energy and carbohydrate intake were 48.2 kcal.kg^−1^.d^−1^ and 8.7 g.kg^−1^.d^−1^, respectively among male players which is higher than female players. However, daily protein and fat intake were higher in female players (1.7 and 1.6 g.kg^−1^.d^−1^, respectively). Both calcium (375.3 mg) and iron (9 mg) intake among Taekwondo players were significantly lower than current sports nutrition guidelines. Nutritional knowledge score (*r* = 0.117), height (*r* = 0.538), weight (*r* = 0.651), body mass index (*r* = 0.347), fat (*r* = 0.075), and energy (*r* = 0.127) intake showed significant positive correlation with strength performance of athletes. The strength performance was positively associated with training hours per day (β = 0.41, 95% CI: 0.09–0.91), body mass index (β = 0.35, 95% CI: 0.09–0.61), nutrition knowledge score (β = 0.13, 95% CI: 0.01–0.25), and energy intake (β = 0.13, 95% CI: 0.12–0.14).

**Conclusions:**

The nutritional knowledge and practice both were suboptimal among Taekwondo athletes. Height, weight, body mass index, nutritional knowledge, energy, and fat intake showed a positive correlation with strength performance. Future studies can build on the premise of this study to identify the robust relationship between nutritional knowledge, practice, different supplement use, and nutrient intake among other athletes too.

## Introduction

The basis of physical performance is nutrition, which provides the energy for biological processes as well as the molecules needed to extract and utilize the potential energy in food (1). For athletes to reach their highest levels of performance, proper nutrition is crucial. As a result, the nutritional status of sports personnel greatly influences their level of physical fitness and training (2). Likewise, good nutritional knowledge and optimum nutrient intake have been regarded as the essential elements that fundamentally contribute to improving sports performance (2). The nutritional status of the Taekwondo (TKD) players is crucial for performance because of weight class sport (3). The adequate nutritional knowledge of TKD athletes is crucial for their ability to cope with the stressful situations they may experience physically, physiologically, and mentally, particularly during training and competitions (4). Therefore, to attain the desired success and performance, TKD athletes should focus on nutritional requirements, adequate energy intake, fluid balance intake, and meal time (4, 5).

Athletic performance can be influenced by a wide range of elements, including physical fitness, practice, diet, and recovery time. Among these, diet plays a crucial role in practice and performance (6). Adequate nutrition has not only a leading role to meet the player's optimum sports performance but also has a crucial role in improving their physical, technical, and tactical training (7, 8). The joint position statement authored by the Academy of Nutrition and Dietetics (AND), the Dietitians of Canada (DC), and the American College of Sports Medicine (ACSM), stresses the critical role of optimal nutrition in athletic performance and recovery from exercise (5). A balanced diet as well as adequate nutritional knowledge are thought to be the essential components that significantly improve athletic performance (9, 10). Incomplete and inadequate information provided by family, teammates, and coaches can also contribute negatively to the athlete's performance (11). In addition, insufficient diet intake and excessive energy expenditure could lead to relative energy deficiency in sport (RED-S) among athletes (12). The RED-S could be exacerbated by poor nutrition knowledge and attitudes toward eating behavior, ultimately diminishing athletic performance (13). Previous studies have shown that athletes who have adequate nutrition knowledge are more likely to meet optimum nutrition requirements (14–16). Inadequate nutrition knowledge can significantly affect athletes' nutritional status and performance, even though results on nutrition knowledge have been contradictory (17, 18).

Previous research has demonstrated the impact of nutrition on a plethora of variables, including health, body composition, substrate availability during activity, recovery following exercise, and critical components for peak athletic performance (5). Good nutrition predicts the ability to train intensely, muscle recovery, and metabolic adaptations for athletes (8). A balanced diet is critical that entails good nutrition management, including the regulated intake of carbohydrates, fats, protein, vitamins, minerals, and water (19). Optimal dietary carbohydrate intake needs to maintain blood glucose levels during exercise and replace muscle glycogen (5). High-quality protein is needed to support muscle protein synthesis, reduce muscle protein breakdown, and repair muscle damage (20). In addition, the optimal timing of protein intake should be considered when determining and prescribing protein needs because it can lead to speedier recovery times and greater training adaptation (19). A certain amount of fat must be consumed to maintain energy balance, promote fatty acid and fat-soluble vitamin intake, sustain overall health, and replenish intramuscular triacylglycerol reserves (19, 21). Fluid intake is recommended at a rate of 0.5–2 L/h, which needs to maintain fluid balance and prevent dehydration. Specifically, fluid intake should be frequent (every 5–20 min) with smaller amounts (150–200 ml) per episode (19). Optimal micronutrients are essential for athletes in line with their metabolic demands. An increase in hemoglobin production, blood volume, and muscle mass are normal characteristics of growth and maturation and account for the majority of amplified iron needs (21, 22). Likewise, with or without iron deficiency can impair muscle function and work capacity, leading to compromised training adaptation and athletic performance (23). Supplementation may be individually prescribed for certain athletes, who have special dietary restrictions or requirements such as vegetarians, and those recovering from injury, or those suffering from a special medical condition (5).

There is a paucity of evidence-based studies exploring factors affecting nutritional knowledge, practice, different supplement use, and nutrient intake with performance among TKD players in Nepal. In addition, sports nutrition knowledge, practice, and nutrient intake among athletes are still unknown so far in the Nepalese context. To the best of our knowledge, this study, for the first time, has explored nutrition knowledge, practice, and nutrient intake with strength performance among Taekwondo players in Nepal. The objective of this study was to generate evidence on nutritional knowledge, practice, the prevalence of supplement use, nutrient intake, and associated strength performance among TKD players.

## Materials and methods

### Study design and setting

This was a cross-sectional study conducted among TKD players of Kathmandu Metropolitan City, Nepal between August 2019 and January 2020. Kathmandu is also the capital and largest city of Nepal and hosts 20% of the country's urban population (24). The city is the center for the majority of sports schools, and 50 out of 85 Taekwondo schools in the country are located within this area (25).

### Sample size, sampling strategy

The sample size was calculated using the single population proportion formula: *n* = *Z*^2^pq/d^2^ (26), taking a 95% confidence interval (CI), 50% proportion (p) for the unknown prevalence of nutritional knowledge or practice among TKD players in the study area, and 6% margin of error (d). Assuming a 10% non-response rate, the total calculated sample was 293.

A two-stage cluster random sampling technique was used to select the desired number of respondents for the study. In the first stage, we considered each of the 35 wards within Kathmandu Metropolitan City as an individual cluster, and then we randomly selected 12 clusters out of 35 clusters. In the second stage, we randomly selected 24 Taekwondo schools out of 50 Taekwondo clubs in Kathmandu valley through the lottery methods. The desired number of participants was then chosen from the selected TKD clubs using probability proportion to size (PPS) methods. Following that, after obtaining written informed consent, interested Taekwondo players from each club were invited to participate in the study and an interview was conducted with those who met the inclusion criteria. The list of schools was obtained from the Nepal Taekwondo Association (NTA) website (25). The athletes who were actively involved in the Taekwondo game and aged 15–25 years were included in the study. Taekwondo players who were absent on the day of the data collection, who refused to participate and those who had special medical conditions such as critical illnesses during the time of data collection were excluded from the study.

### Data collection and study variables

#### Predictor variables

Data were collected by trained undergraduate public health students who were provided with 3 days of training that included the objective of the study, data collection procedure, sampling method, ethical aspects of the study, and data entry techniques. Face-to-face interviews were conducted using pre-tested semi-structured questionnaires. Interviews were conducted at the Taekwondo School's premises or the training grounds at least 45 min before or after the training. All the tools were originally developed in the English language. Further, the tools were translated into the Nepali language and back-translated into English to ensure the validity (and reliability) of the tool. Pretesting of the tools was carried out among 30 Taekwondo players from the clubs located in the areas of Lalitpur Metropolitan City. The research committee, faculty members, and dietitians reviewed the pre-tested questionnaire to establish its validity and reliability of the questionnaire. The questionnaire was revised appropriately based on their feedback. The questionnaire consisted of four parts:

##### Socio-demographic and behavioral information

Information on participants' age, sex, ethnicity, family type, education, household monthly income, sources of information, and consumption of alcohol and tobacco use were collected. The participant's ethnicity was categorized into advantaged and disadvantaged ethnic groups for further analysis. The family type was categorized into nuclear and joint families. A joint family typically consists of three or more generations and their spouses living together as a single household and a nuclear family refers to a two-generation family consisting of a father and mother and children or a single, possibly widow, parent and his/her children (27).

##### Anthropometric measurements

Height, weight, and body mass index (BMI) according to WHO classification. The anthropometric measurements including the weight and height of the participants were measured according to standardized procedures. Weight was measured to the nearest 0.1 kg using a digital weighing machine three times, and the average values were documented. Height was measured in the standing position to the nearest 0.1 cm with a portable stadiometer three times with 1-min rest in between and the mean value was documented. The BMI was calculated using weight (kg) divided by height squared (m^2^) and categorized using the World Health Organization (WHO) cut-off points for BMI (28).

##### Nutrition knowledge and practice

Questionnaires on nutritional knowledge and practice and procedure used for the classification were adapted from previous works (16, 29, 30) (Supplementary material: Questionnaire). There were 22 statements in the knowledge section that could be answered as “true” or “false.” Each correct response received a “1,” while incorrect responses received a “0.” In the practice section, 15 statements with “yes” or “no” responses were prepared. Each positive response in this section received a “1,” while each negative response received a “0.” The median score was used as the cutoff point to assess whether a person had good or bad knowledge and practice scores. Despite criticism, the median split can produce the best results when the variable is continuous and normally distributed (31). After that, based on the participant's responses, the median nutrition knowledge (NK) score was determined. Participants were categorized as having poor knowledge if their scores were below the median nutrition knowledge score or as having good knowledge if their scores were equal to or higher than the median nutrition knowledge score. The scoring process for NP and categorizing poor NP and good NP were similar to the procedure used for the classification of nutrition knowledge. The Cronbach's alpha of nutrition knowledge and practice questionnaire and had found acceptable consistency of 0.79, and 0.75, respectively.

##### Dietary intake

Pre-tested 24-h dietary recall tool, based on Nepalese food and beverage was used to assess the previous 24 h (midnight to midnight) nutrient intake of the participants (32). During 24-h recall, the athletes were asked to name all the food and drink items consumed during the preceding day, including anything consumed outside the home and the time of consumption was also recorded. If multiple servings of the same food items were reported to be consumed in a single eating occasion, then these amounts were combined into a single portion. The portion size of items consumed was estimated using a graduated measuring cylinder and standard weight for foods that are served as a unit (boiled egg, bread slice), as per the principle and guideline of the Indian Institute for Medical Research (ICMR) (33). Based on the information obtained from the 24-h dietary recall method, the number of foods was then converted into daily nutrient intakes. The mean daily intake of total energy, carbohydrates, protein, fats, calcium, and iron over 24-h recalls was calculated accordingly using the Nutrition Society of India (NSI) diet calculator developed by the National Institute of Nutrition, ICMR, Hyderabad, India (34) and food compostion table, Nepal (35). The mean daily energy, protein, carbohydrate, and fat intake was compared with the values reported in the Nutrition and Hydration Guidelines for Excellence in Sports Performance, National Institute of Nutrition, India (36) and current American College of Sports Medicine (ACSM) sports nutrition guidelines (5). The macronutrients were reported in grams (g), relative to body weight (BW) (g·kg·BW^−1^) while micronutrients were expressed in milligrams (mg). Nutrition and Hydration Guidelines states have categorized weight division games such as TKD under Group-V sports events which demand a minimum of 3,700 Kcal of daily energy, which means 55–65%, 12–15%, and 25–30% should be yielded from carbohydrate, protein and fat, respectively (36). The American College of Sports Medicine's (ACSM) sports nutrition guidelines state that the daily protein intake recommendations for sport nutrition range from 1.2 to 2 g/kg.BW/day while the daily carbohydrate intake recommendations range from 3 to 12 g/kg.BW/day (5).

##### Dietary supplement

Information on dietary supplements used by TKD players was collected using a semi-structured questionnaire. A dietary supplement is a commercially available product that is consumed as an addition to the usual diet including vitamins, minerals, herbs, amino acids, and a variety of ergogenic compounds (37).

#### Outcome variable

##### Strength performance

Handgrip strength (HGS), as an indirect measure of strength performance, was assessed with the handgrip dynamometer (16, 38–40). Hand grip strength is considered one of the important variables that determine physical fitness in Taekwondo athletes (41). Measurements were performed for both hands, and handedness (dominance and non-dominance) was determined based on self-report and the dominant handgrip strength (DHGS) (16, 38). Handgrip strength was measured using a standard adjustable digital handgrip dynamometer (Hand Grip Dynameters Labappara, Ambala Cantt, Haryana of India) at the standing position with the shoulder adducted, neutrally rotated, and elbow in full flexion. The dynamometer was held freely without support. The participants were asked to squeeze the handle maximally and to sustain it for 3–5 s. All measurements were performed for both hands, and measurements were performed thrice for each hand, and the mean value was documented. A 1-min rest was provided between each attempt, and hands were alternated to minimize fatigue effects. The results were documented in kilogram-force (kgf). The handgrip dynamometer was calibrated before each assessment.

### Data management and analysis

Before entering data for analysis, data were manually checked and compiled to ensure completeness and accuracy. The collected data were entered into EpiData software 3.1v and transferred into Stata/MP version 14.1 (StataCorp LP, College Station, Texas) for statistical analysis. The descriptive data were presented in the form of mean, standard deviation, frequency, and percentage for normally distributed data. Pearson's chi-square (χ2) was applied for categorical variables, and for continuous data, independent sample *t*-test and one-way analysis of variance (ANOVA) tests were performed to observe differences in socio-demographic characteristics and strength performance. One sample *t*-test was used to assess the macro and micronutrient intake compared to the corresponding RDA of athlets. Pearson correlation and simple linear regression analysis were used to observe the relationship between the predictor and outcome variables. The statistical significance was considered at *p*-value <0.05 and 95% confidence intervals (CIs).

### Ethical considerations

The ethical approval for this study was obtained from the ethical review board of the Nepal Health Research Council (Reference number 53/2019). Formal permission was also obtained from the respective Taekwondo schools. Written informed consent was obtained from all parents or legal guardians for eligible participants who were aged <18 years and from participants themselves who were aged >18 years before proceeding to data collection. We have also taken informed consent from the participants below 18 years using an assent form. The data collectors shared the objectives of the study with athletes and coaches of respective schools before the data collection. Information was provided to participants regarding their freedom to refuse participation at any time and the confidentiality of their identity.

## Results

### Characteristics of the participants

**Figure 1:**
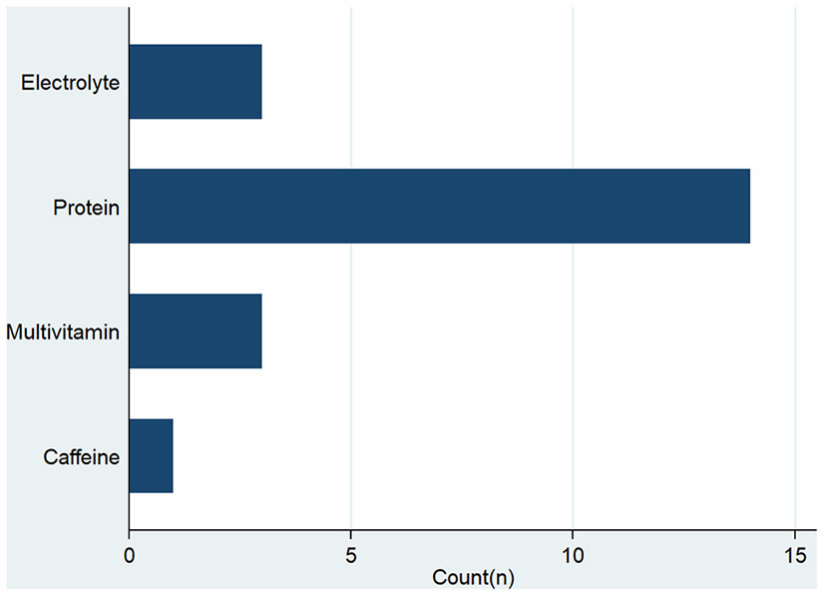
Type of dietary supplements used by Taekwondo players.

A total of 293 participants were included in this study. The mean (±SD) age of the participants was 18.0 (±3.2) years, and more than two-thirds (70.9%) of participants were younger (15–19 years) age groups. Nearly two-thirds, 63.1% (185/293) were male and the rest were female. More than half of the participants, 55.4% (161/293) had completed a higher secondary or higher level of education (University level of education) while 45% had completed a secondary or lower level of education. About half of the participants, 60.7% (178/293) were engaged in <2 h of training per day. The majority of the participants did not smoke, 97.6% (286/293), and more participants, 97.6% (286/293) did not consume alcohol (Table 1). The majority of the participants, 92% (272/293) did not use any dietary supplements (Figure 1).

**Table 1:** Socio-demographic and anthropometric characteristics with DHGS score of the study participants (*n* = 293).

**Variables**	**Characteristics**	***n* (%)**	**DHGS score mean ±SD**	***p*-value^A,B^**
Age (years)	–		18.0 ± 3.1	–
Age categories	15–19	208 (70.9)	29.5 ± 1.1	<0.001^*^
	20–25	85 (29.1)	42.1 ± 1.7	
Sex	Male	185 (63.1)	38.3 ± 17.3	<0.001^*^
	Female	108 (36.8)	24.1 ± 11.1	
Ethnicity	Advantaged ethnic groups	131 (44.7)	33.1 ± 17.2	0.532
	Disadvantaged ethnic group	162 (55.3)	33.2 ± 30.6	
Education level	Secondary or lower	132 (45.1)	27.9 ± 16.4	<0.001^*^
	Higher secondary or above	161 (54.9)	37.4 ± 15.8	
Occupation	Students	221 (75.4)	29.5 ± 14.8	<0.001^*^
	Service	72 (24.5)	44.1 ± 17.5	
Monthly family income^a^ (NRs) (1 USD = 110 NRs)	≤30 k	157 (53.6)	32.4 ± 16.4	0.310
	>30 k	136 (46.4)	34.6 ± 17.5	
Training hours per day^a^	≤2 h	178 (60.7)	29.6 ± 14.6	<0.001^*^
	>2 h	115 (39.3)	42.6 ± 18.5	
Smoking habit	Yes	7 (2.4)	42.4 ± 18.2	0.138
	No	286 (97.6)	32.9 ± 16.7	
Consumption of alcohol	Yes	7 (2.4)	48.2 ± 15.4	0.015^*^
	No	286 (97.6)	32.7 ± 16.6	
Family type	Nuclear	165 (56.3)	32.5 ± 16.9	0.501
	Joint	128 (43.6)	33.8 ± 16.6	
BMI (kg/m^2^)			20.3 ± 2.5	–
	Underweight (< 18.5)	56 (19.1)	24.4 ± 11	<0.001^*^
	Normal (18.5–24.9)	231 (78.8)	35 ± 17.2	
	Overweight (≥25–29.9)	6 (2.1)	42.6 ± 18.7	

A,B Independent sample t-test and one-way ANOVA were used for DHGS;

* denotes statistically significant at *p* < 0.05; DHGS, dominant handgrip score; NRs, Nepalese rupees;

a Median split was used to dichotomize the variable.

Out of 293 participants, 54.3% (159/293) participants had poor nutrition knowledge, and 55.3% (162/293) participants had poor nutrition practice scores. The mean (±SD) scores of NK and NP were 10.1 (±2) and 9.0 (±2.3), respectively. The mean (±SD) score of the right handgrip strength score was 35.4 (±16.9), while the left handgrip strength score was 30.8 (±17.6) (Table 2).

**Table 2:** Nutrition related information of study participants with dominant hand grip strength (*n* = 293).

**Variables**	**Characteristics**	***n* (%)**	**DHGS score mean ±SD**	***p*-value^a,b^**
Nutrition knowledge score	Poor	159 (54.3)	34.2 ± 17.2	0.305
	Good	134 (45.7)	36.3 ± 16.8	
Nutrition practice score	Poor	162 (55.3)	33.8 ± 16.8	0.174
	Good	131 (44.7)	36.5 ± 17.2	
Sports supplements use	Yes	21 (7.2)	30.9 ± 16.6	0.530
	No	272 (92.8)		
Total nutrition knowledge score	–	–	10.12 ± 3.94	–
Total nutrition practice score	–	–	9.01 ± 2.35	–
Right handgrip strength score (kgf)	–	–	35.4 ± 16.9	–
Left handgrip strength score (kgf)	–	–	30.8 ± 17.6	–

a,b Independent sample t-test and one-way ANOVA were used for DHGS; DHGS, dominant handgrip score.

The males had poor nutrition knowledge (66.7%) and practice score (61.1%) compared to females. Likewise, the majority of younger players had poor nutrition knowledge (71.7%) and poor practice score (72.2%) compared to the age group of 20–25 years. There was no significant different between NK score (52.7%) and NP (55.6%) (Table 3).

**Table 3:** Association of age, gender with nutrition knowledge and nutrition practice among Taekwondo players (*n* = 293).

**Variables**	**Nutritional knowledge**		***p*-value^a^**	**Nutritional practice**		***p*-value^b^**
	**Good *n* (%)**	**Poor *n* (%)**		**Good *n* (%)**	**Poor *n* (%)**	
**Sex**						
Male	79 (58.9)	106 (66.7)	0.173	86 (65.6)	99 (61.1)	0.423
Female	55 (41.1)	53 (33.3)		45 (34.4)	63 (38.9)	
**Age group**						
15–19	94 (70.2)	114 (71.7)	0.771	91 (69.5)	117 (72.2)	0.605
20–25	40 (29.8)	45 (28.3)		40 (30.5)	45 (27.8)	
**Nutrition knowledge**						
Good	–	–	–	62 (47.3)	72 (44.4)	0.622
Poor	–	–	–	69 (52.7)	90 (55.6)	

a Chi squared test was applied for sex and age group with nutrition knowledge score;

b Chi-squared test was applied for sex, age and nutrition knowledge with nutrition practice score.

### Nutrient intakes among players

The mean (SD) total energy intake was 2397.2 (534.5) kcal/day, while male players consumed 2405.3 (545.7) kcal/day compared to female players (2383.2 kcal/day). The daily mean energy intake was 48.0 kcal/kg/ body weight, whereas male players consumed 48.2 kcal.kg^−1^.d^−1^ and female players consumed 47.6 kcal.kg^−1^.d^−1^. The mean (SD) intake of carbohydrates, protein, and fat was 430.5 (124.9), 83.7 (23.7), and 76.4 (27.6) g, respectively. Daily mean carbohydrate protein and fat intake were 8.6, 1.6, and 1.5 g.kg^−1^.d^−1^, respectively, which is within the recommended limit of RDA. The mean intake of carbohydrates by male players was 8.7 g.kg^−1^.d^−1^ which is higher than that of female players (8.4 g.kg^−1^.d^−1^). Interestingly, the mean daily intake of protein and fat was 1.7 and 1.6 g.kg^−1^.d^−1^ among female players which is higher than among male players. However, iron consumption was less in female (8.3 mg.kg^−1^.d^−1^), while male players consumed 9.4 mg.kg^−1^.d^−1^. The mean (SD) calcium and iron intake were 375.3 mg (235.5 mg) and 9 mg (7.1 mg), respectively. All these macro and micro nutrients were significantly lower than the corresponding RDA of players recommended by the Nutrition and Hydration Guidelines for Excellence in Sports Performance, National Institute of Nutrition, India, and current American College of Sports Medicine (ACSM) sport nutrition guidelines (Table 4).

**Table 4:** Description of daily nutrient intake per day compared to corresponding RDA of Taekwondo players (*n* = 293).

**Nutrients**	**Male players mean (SD)**	**Female players mean (SD)**	**Total players mean (SD)**	**Recommendation**	***p*-value^†^**
Energy (kcal)	2405.3 (545.7)	2383.2 (517.9)	2397.2 (534.5)	3,700^a^	<0.001^*^
Energy kcal.kg^−1^.d^−1^	48.2 (14.1)	47.6 (14.7)	48.0 (14.3)	60^a^	<0.001^*^
Carbohydrate (g)	435.1 (126.5)	422.5 (122.4)	430.5 (124.9)	500^a^	<0.001^*^
Carbohydrate g.kg^−1^.d^−1^	8.7 (3.4)	8.4 (3.1)	8.62 (3.1)	6–10^b^	<0.001^*^
Protein (g)	83.4 (24.4)	84.3 (22.5)	83.7 (23.7)	148^a^	<0.001^*^
Protein g.kg^−1^.d^−1^	1.6 (0.6)	1.7 (0.6)	1.6 (0.6)	1–2^b^	<0.001^*^
Fat (g)	76.1 (28.5)	77.1 (26.2)	76.4 (27.6)	125^a^	<0.001^*^
Fat g.kg^−1^.d^−1^	1.5 (0.6)	1.6 (0.7)	1.5 (0.6)	2.1^a^	<0.001^*^
Calcium (mg)	364.2 (230.0)	394.2 (244.6)	375.3 (235.5)	1,000^b^	<0.001^*^
Iron (mg)	9.4 (7.1)	8.3 (6.8)	9.0 (7.1)	18^b^	<0.001^*^

a Recommended dietary allowance for weight division category group-V of athletes;

b RDA of current American college of sports medicine (ACSM) sports nutrition guidelines;

† One-sample t-test;

* Statistically significant at *p* < 0.05.

### Correlation of strength performance with different covariates

Table 5 presents the correlation of anthropometric measurements, nutrition knowledge, practice, supplement use, and nutrient intake with strength performance among Taekwondo players. Various factors were found to have a positive correlation with strength performance. Nutritional knowledge score (*r* = 0.117, *p* < 0.045), height (*r* = 0.538, *p* < 0.001), weight (*r* = 0.651, *p* < 0.001), BMI (*r* = 0.347, *p* < 0.001), fat (*r* = 0.075, *p* < 0.002), and energy (*r* = 0.127, *p* < 0.029) intake were positively correlated with strength performance of athletes (Table 5).

**Table 5:** Correlation of anthropometric measurements, nutrition knowledge, practice, and nutrient intake with strength performance among Taekwondo players (*n* = 293).

**Variables**	**DHG score**	
	**Pearson correlation (*r*)**	***p*-value**
Height (feet)	0.538	0.001^*^
Weight (kg)	0.651	0.001^*^
BMI (kg/m^2^)	0.347	0.001^*^
NK score	0.117	0.045^*^
NP score	0.008	0.957
Supplements use	0.050	0.387
Carbohydrate intake (g)	0.085	0.145
Protein intake (g)	0.128	0.608
Fat intake (g)	0.075	0.002^*^
Calcium intake (mg)	0.054	0.355
Iron intake (mg)	−0.004	0.994
Energy (kcal)	0.127	0.029^*^

* Statistically significant at *p* < 0.05; DHG score, dominant handgrip score; NK, nutrition knowledge; NP, nutrition practice.

Table 6 presents the linear regression analysis of anthropometric measurements, nutrition knowledge, practice, and nutrient intake with strength performance among Taekwondo players. The DHGS was positively associated with training hours per day (β = 0.41, 95% CI:−0.09–0.91, *p* < 0.001), BMI (β = 0.35, 95% CI: 0.09–0.61, *p* < 0.001), NK score (β = 0.13, 95% CI: 0.01–0.25, *p* = 0.024), and energy intake (β = 0.13, 95% CI: 0.12–0.14, *p* < 0.035). About 23.7% of the variance in the DHGS, which is the measure of strength performance is explained by this model (Table 6).

**Table 6:** Linear regression analysis of anthropometric measurement, nutrition knowledge, practice, and nutrient intake with strength performance among Taekwondo players (*n* = 293).

**Variables**	**β (95% CI)**	**SE**	***p*-value**
Training hours per day	0.41 (−0.09–0.91)	0.61	<0.001^*^
BMI (kg/m^2^)	0.35 (0.09–0.61)	0.37	<0.001^*^
Nutrition knowledge score	0.13 (0.01–0.25)	0.47	0.024^*^
Nutrition practice score	−0.03 (0.001–0.061)	0.53	0.543
**Nutrient intake**			
Energy (kcal)	0.13 (0.12–0.14)	0.001	0.035^*^
Carbohydrate (g)	0.05 (0.04–0.052)	0.002	0.343
Protein (g)	0.13 (0.12–0.14)	0.01	0.124
Fat (g)	0.11 (0.10–0.12)	0.007	0.054
Calcium (mg)	0.05 (0.04–0.06)	0.004	0.425
Iron (mg)	0.02 (0.01–0.03)	0.13	0.667

Adjusted R-squared = 0.237, *F*_(10, 282)_ = 10.09, *p* < 0.001.

* Statistically significant at *p* < 0.05.

## Discussion

This may be the first study of its kind to assess the association of nutrition knowledge, practice, and nutrient intake and supplementation use with strength performance among Taekwondo players in Nepal. Good nutritional knowledge, practice, and adequate nutrient intake are considered crucial components contributing to strength performance (42).

In this study, based on the correct response of the nutrition knowledge and practice score, the study participants demonstrated a relatively low level of nutrition knowledge and practice compared to previous findings where less than half had poor nutritional knowledge (16, 43, 44). The optimum nutrition knowledge is crucial for fostering athletic performance in athletes, which further shows that increasing nutrition knowledge is related to dietary intake and subsequent sports performance (10, 45). In addition, systematic review has shown that athletes who obtain nutrition education might become more understanding about nutrition, which will optimize their dietary intake (18). In contrast, a previous systematic review related to athletes' have found that the nutrition knowledge of athletes and its effect on their dietary intake is equivocal. This can be the result of errors in the NK measurement and a lack of validation (17). Inadequate nutrition knowledge can have a serious impact on the nutritional status and performance of athletes (2). The study conducted by Walsh et al. (13) showed that poor nutritional knowledge and attitude contribute to poor dietary behaviors. This study revealed that those athletes who had poor nutrition knowledge were found to have poor nutrition practice scores. These findings were in line with previous findings where the correlation between nutrition knowledge and the practice score of athletes was statistically significant (16, 46). Lack of knowledge about nutrition in sports is considered one of the potential barriers to optimal nutrition practices among athletes (15).

The prevalence of supplement use in the current study was found to be lower than that reported in other studies (9, 47). Overall, 7.2% of Taekwondo players in our study used some form of supplement (Figure 1). Likewise, Muwonge et al. (48) have reported the prevalence of supplement use as 13.4%, which is also low compared to previous study findings (4, 9, 49). This discrepancy is due to different sports categories, country contexts, and variable sample sizes involved in other studies. Professional TKD players may use nutritional supplements mainly to maintain their good health and nutritional status to improve strength performance.

Every athlete including TKD players needs adequate calories from the diet in terms of optimum quality and quantity before, during, and after exercise for improved athletic performance (8). In this study, daily carbohydrate intake was 8.6 g.kg^−1^.d^−1^, while male players had consumed 8.7 g.kg^−1^.d^−1^ and female players 8.4 g.kg^−1^.d^−1^. The amount of carbohydrates consumed in this study is larger than the previous findings among Korean Taekwondo players (6). According to the findings of the systematic review, athletes consumed substantially less carbohydrates (2.4–6 g/kg.bw/d) than the average person (17). This can be because the amount of carbohydrates needed varies depending on the type of sport. The recommended daily carbohydrate intake for athletes ranges from 3 to 12 g/kg/BW/day, whereas endurance programs involving 1–3 h of moderate- to high-intensity activity require 6–10 g/kg.BW/day (5). Similarly, the National Institute of Nutrition in India's Nutrition and Hydration Guidelines for Excellence in Sports Performance recommended 8.3 g/kg/BW/day of carbohydrates for the group-V sports category (34). TKD players' carbohydrate consumption is sufficient when compared with current sports guidelines in terms of relative nutritive value. Carbohydrate is essential not only as a source of energy but also protects the protein from being exploited as an energy source (50, 51). Inadequate intake of carbohydrates increases the risk of injury and exaggerates the deterioration of athletic performance (51, 52). Thus, carbohydrate intake before, during, and after exercise should be scaled up according to the characteristics of the event. Even small amounts of carbohydrate solution obtained from mouth-rinsing without swallowing increases athletic performance through the pathway that involves the central nervous system in its absorption (50).

In this study, male players consumed 1.6 g.kg^−1^.day of protein on average per day, whereas female players consumed 1.7 g.kg^−1^.day of protein every day. Protein intake was consistent with current recommendations (1.2–2 g·kg^−1^·day) (5) and consistent with previous findings (6, 52). Inadequate protein consumption can have serious health problems in athletes such as the inability to maintain proper body function and a decline in sports performance (21). A previous study showed that a protein intake of 1.4–2.0 g/kg body weight could improve body adaptability for intensive physical activities (53). Adequate protein intake is critical in the overall exercise training program, required for proper and speedy recovery from injury including bolstering immune function, growth, and maintenance of lean body mass (53).

The mean fat intake was found to be low (76.4 g) in this study. Nutrition and Hydration Guidelines for Excellence in Sports Performance, India recommended fat intake is 148 g for Group-V sports events (36). In terms of fat intake with body weight, male players had a daily intake of 1.5 g/kg/BW and female players had a daily intake of 1.6 g/kg/BW, which is less than the corresponding RDA of 2.1 g/kg/BW (36) and is in agreement with a recent study among Taekwondo players in Korea (6). On the other hand, other studies have found that fat intake was found to have higher than the corresponding RDA (52, 54). Fat is an essential component of a balanced diet, providing energy, optimum elements of cell membranes, and facilitating the absorption of fat-soluble vitamins (2).

The mean energy intake in these participants was 2397.2 kcal/day, which is lower than the corresponding RDA and slightly lower than reported in the previous study in Indian Taekwondo athletes (the mean energy intake was 3,129 ± 518.9) (3). The average daily energy consumption for male players was 48.2 kcal/kg/BW, whereas it was somewhat lower for female players (47.6 kcal/kg/BW). This finding is consistent with previous studies among TKD players in Korea (6) and Football players in Australia (52).

The mean intake of micronutrients: calcium (375.3 mg) and iron (9 mg) were below the current sport nutrition guidelines (5, 36). These findings are in line with the results from the other studies (3, 52), where their daily intake was also below the RDA. Male athletes' daily iron intake was somewhat greater (9.4 mg/day) than that of female athletes (8.3 mg/day). However, these values are less than current sport nutrition guidelines (5, 36) and consistent with previous study conducted among female athletes in Australia (52). Korean TKD athletes consumed an inadequate amount of calcium (6), which is consistent with our findings. However, compared to our study, the iron intake of Korean TKD athletes was higher (6). Adequate intake of iron, which is necessary to carry oxygen, and maintain energy production, is critical for female athletes during high-intensity exercise, owing to their iron loss during the regular menstrual cycle (55). In addition, a sufficient amount of calcium-rich diet contributes to bolstering bone mineral density and preventing fall injury and fracture (52).

This study shows the highest gap in meeting the nutrient requirement in iron intake and the least in carbohydrate intake. This may be due to the typical Nepalese dietary pattern (56, 57) where carbohydrate-rich food (rice, chapattis, bitten rice) is overemphasized and predominant while lack in iron-rich food like green leafy vegetables, various fruit, and animal source food (meat, egg, milk). On the other hand, Taekwondo is a weight-classified combat sport and it's very common for an athlete to try to lose weight before competition by dieting, fasting, or fluid restriction (58, 59) which might have been one other reason for inadequate nutrient intake. But we couldn't assess it in our recent paper.

Anthropometric characteristics such as height, weight, and BMI were positively correlated with strength performance. These results are consistent with the previous studies where they reported that handgrip strength had a strong correlation with various anthropometric characteristics (16, 38). In this study, results indicated a significant relationship between NK scores and strength performance. Various previous studies reported that nutrition knowledge was positively correlated with athletic performance (13, 16, 46). Higher nutrition knowledge of the athletes, better their attitude and practice toward a sport-enhancing diet, resulting in better performance (46). Likewise, total energy and fat intake were significantly positively correlated with the handgrip strength of athletes. The previous study also reported that energy intake was positively associated with handgrip strength (16). The most important component to optimize athletic performance through diet is to ensure that the athlete is consuming enough calories to maintain a positive energy balance (7, 19). The large volume of rigorous exercise, meanwhile, may make it difficult to meet the energy requirements of athletes. Inadequate energy intake can lead to injury, illness, increased prevalence of overtraining syndrome, weight loss, particularly of lean muscle mass, and finally impaired exercise performance (60). Moreover, in linear regression analysis, the strength performance was positively associated with training hours per day, BMI, NK score, and energy intake. These results are consistent with Folasire et al. (16) findings. Along with injury prevention and risk assessment for health, optimum anthropometric measures may also help improve strength performance. The ideal body weight based BMI and the ideal body fat percentage should be taken into consideration when determining the optimum anthropometric measurement (61). Previous studies have shown that higher dietary intake is associated with higher athletic performance (62, 63). Nutrition knowledge is one component that may improve dietary intake (17) and subsequently enhance athletic performance (39). Individuals are thought to be more successful in sports life if they understand the significance of an adequate and balanced diet and use this information in their habits (30).

Our study had a few limitations. The sample size used in this study was confined to Taekwondo players, thus we cannot generalize the findings to all types of sports. There are significant differences in nutritional requirements depending on the type of sport. This study used a single 24-h recall method to obtain the nutrient intake, which does not accurately reflect the respondents' usual dietary habits and nutrient intake levels. Also, the software used to calculate the micronutrient did not have sufficient data for all food items, and can not identify within and between-person variations. Although other minerals and vitamins play a key role in human health and nutritional status, we included only calcium and iron as micronutrient intake. Since the information was collected through face-to-face semi-structured questionnaires, there may have been social desirability bias. Furthermore, we did not collect data on the training cycle to determine whether TKD players were in the weight-cutting phase. Due to the limited resources, we could not measure the body composition using a portable body composition device and also could not assess the other anthropometric measurements such as waist, neck, and hip circumference. Despite the mentioned limitations, our study has some strengths. Our study included an analysis of the correlation between nutritional knowledge, practice, and nutrient intake with strength performance. This evidence provide insight into the potential factors influencing strength performance which helps in forming the basis of targeted nutrition education programs. The majority of the national Taekwondo clubs were located in Kathmandu Metropolitan City, however, the study sample included players from all levels of Nepal. The results of this study can be a reference to reform the nutritional policy for all types of athletes. This study has also set the premise to build further evidence to explore the determining factors for nutritional knowledge and practices among Nepalese athletes.

## Conclusions

The study shows the both nutrition knowledge and practice were poor among Taekwondo players in Nepal. Total energy intake, protein, fat, calcium, and iron intake were low against the current recommendations for athletes. BMI, nutritional knowledge, energy, and fat intake were positively associated with strength performance. Future research is essential to explore the relationship between nutritional knowledge, practice, nutrient intake, and dietary supplement use for strength performance among athletes of different sports. Integrating nutrition expert and their recommendations in sports is essential to ensure that athletes are on an optimal diet so that it can ultimately enhance their strength performance.

## Data availability statement

The original contributions presented in the study are included in the article/Supplementary material, further inquiries can be directed to the corresponding author/s.

## Ethics statement

The studies involving human participants were reviewed and approved by Ethical Review Board (ERB) of Nepal Health Research Council. Written informed consent to participate in this study was provided by the participants' legal guardian/next of kin.

## Author contributions

DSu: research design, the conceptualization of the idea, methodology, formal data analysis, interpretation, software, validation, writing an original draft, writing review, editing, and overall supervision of the research. DSi: research design, the conceptualization of the idea, methodology, data analysis, interpretation, writing an original draft, writing review, editing, and overall supervision of the research. MB: research design, data collection, data curation, writing an original draft, and writing a review, editing. VS, KK, and PP: writing an original draft, reviewing and editing, and overall supervision of the research. All authors read and approved the final manuscript.

## Conflict of interest

The authors declare that the research was conducted in the absence of any commercial or financial relationships that could be construed as a potential conflict of interest.

## Publisher's note

All claims expressed in this article are solely those of the authors and do not necessarily represent those of their affiliated organizations, or those of the publisher, the editors and the reviewers. Any product that may be evaluated in this article, or claim that may be made by its manufacturer, is not guaranteed or endorsed by the publisher.
