# Supplementary material for: Association of nutrition knowledge, practice, supplement use, and nutrient intake with strength performance among Taekwondo players in Nepal
Source: Front Nutr. 2022 Nov 14;9:1004288. doi: 10.3389/fnut.2022.1004288 (PMC9703343; doi:10.3389/fnut.2022.1004288)
Supplement: Supplementary file 1 [file Table_1.DOCX]

# Association of nutrition knowledge, practice, supplement use and nutrient intake with strength performance among Taekwondo players in Nepal

QUESTIONNAIRE

Participants ID no: …………………….. Date…………………..

District ……………………….

Municipality: …………………………..

**Section A. Socio-demographic Information**

| S.N. | Question | Response | Code | Remarks |
| --- | --- | --- | --- | --- |
| 1 | Age | …….. |  |  |
| 2 | Sex | Male | 1 |  |
|  |  | Female | 2 |  |
| 3 | Religion | Hindu | 1 |  |
|  |  | Buddhist | 2 |  |
|  |  | Christian | 3 |  |
|  |  | Muslim | 4 |  |
|  |  | Others | 5 |  |
| 4 | Ethnicity | Brahmin | 1 |  |
|  |  | Chhetri | 2 |  |
|  |  | Dalit | 3 |  |
|  |  | Janajati | 4 |  |
|  |  | Muslim | 5 |  |
|  |  | Others | 6 |  |
| 5 | Family type | Nuclear | 1 |  |
|  |  | Joint | 2 |  |
| 6 | Education status | Illiterate | 1 |  |
|  |  | Primary | 2 |  |
|  |  | Secondary | 3 |  |
|  |  | Higher secondary | 4 |  |
|  |  | Bachelor or above | 5 |  |
| 7 | Occupation | Student | 1 |  |
|  |  | Agriculture | 2 |  |
|  |  | Service | 3 |  |
|  |  | Labor | 4 |  |
|  |  | Others | 5 |  |
| 8 | Family income | ………. |  |  |
| 9 | Smoking habit | Yes | 1 |  |
|  |  | No | 0 |  |
| 10 | Alcohol habit | Yes | 1 |  |
|  |  | No | 0 |  |
| 11 | Training hours per day | ……… |  |  |
| 12 | Degree of belt | ……… |  |  |
| 13 | Height | ……….. |  |  |
| 14 | Weight | ……….. |  |  |
| 15 | BMI | ………. |  |  |
| 16 | Right handgrip strength score | ……….. |  |  |
| 17 | Left handgrip strength score | ………… |  |  |

**Section B: Nutritional Knowledge**

| **SN** | **Nutrition knowledge questions** | **True** | **False** |
| --- | --- | --- | --- |
|  |  | 1 | 0 |
| 1 | Protein is the main energy source for the muscle |  |  |
| 2 | Fats have important roles in the body |  |  |
| 3 | Iron-deficiency anemia results in a decrease in the amount  of oxygen that can be carried in the blood |  |  |
| 4 | Iron in meat is absorbed at the same rate as iron in a plant  food |  |  |
| 5 | The body can make vitamin D upon exposure to the sun |  |  |
| 6 | Vitamin supplements are recommended for all physically  active people |  |  |
| 7 | During physical activity, feeling thirsty is enough to indicate the need for liquid |  |  |
| 8 | Skipping meals is justifiable if you need to lose weight quickly |  |  |
| 9 | Foods like chocolate, biscuits and chips are the most appropriate foods to be consumed soon after training |  |  |
| 10 | Vitamins are good sources of energy |  |  |
| 11 | Alcohol consumption can affect absorption and utilization of nutrients |  |  |
| 12 | Saturated and unsaturated oils both have an equal effect on the health |  |  |
| 13 | Eating carbohydrates makes you fat |  |  |
| 14 | Dehydration decreases performance |  |  |
| 15 | The last meal before a competition should be eaten 3-4 hours before the competition |  |  |
| 16 | Males and females of the same age group spend equal amounts of calories during the same exercise |  |  |
| 17 | Bananas are good sources of potassium |  |  |
| 18 | Table salt is an essential part of a healthy diet |  |  |
| 19 | Milk and milk products are the best sources of calcium |  |  |
| 20 | Basic sugars like brown or granulated sugar, jam and honey are the most suitable energy sources for sportsmen |  |  |
| 21 | Carbohydrates are stored in muscles in the form of glycogen |  |  |
| 22 | The last meal before a competition should be consumed 3-4 hours before the competition |  |  |

**Section C: Nutritional practice**

| **SN** | **Nutrition practice questions** | **Yes** | **No** |
| --- | --- | --- | --- |
|  |  | 1 | 0 |
| 1 | Do you use supplements like multivitamin as an athlete? |  |  |
| 2 | I consume lots of fruits and vegetables |  |  |
| 3 | I skip meals before a competition or an event |  |  |
| 4 | I eat just before an event |  |  |
| 5 | I eat just after an event |  |  |
| 6 | I consume sports drinks every day during practice or when I feel dehydrated |  |  |
| 7 | I eat adequate diet daily |  |  |
| 8 | I change my pattern of eating at the time of a competition |  |  |
| 9 | I always take my breakfast daily |  |  |
| 10 | I consume lots of water during and after training/competition |  |  |
| 11 | I always eat at least one hour before training/competition |  |  |
| 12 | I prefer snacks to special diet before training and competition |  |  |
| 13 | I eat at least 3 times daily |  |  |
| 14 | I consume milk and milk products daily |  |  |
| 15 | I consume alcohol to enhance my performance |  |  |

**Section D: A 24-hours Dietary Recall**

| **Time** | **Food/drink** | **Amount** | **Household measurement** | **Remarks** |
| --- | --- | --- | --- | --- |
| **Early morning** |  |  |  |  |
|  |  |  |  |  |
|  |  |  |  |  |
|  |  |  |  |  |
|  |  |  |  |  |
| **Breakfast(8:00-8:30 am)** | **Food/drink** | **Amount** | **Household measurement** | **Remarks** |
|  |  |  |  |  |
|  |  |  |  |  |
|  |  |  |  |  |
|  |  |  |  |  |
|  |  |  |  |  |
| **Launch (10:00-11:00am)** | **Food/drink** | **Amount** | **Household measurement** | **Remarks** |
|  |  |  |  |  |
|  |  |  |  |  |
|  |  |  |  |  |
|  |  |  |  |  |
|  |  |  |  |  |
|  |  |  |  |  |
|  |  |  |  |  |
| **Afternoon Tea(3:00pm)** | **Food/drink** | **Amount** | **Household measurement** | **Remarks** |
|  |  |  |  |  |
|  |  |  |  |  |
|  |  |  |  |  |
|  |  |  |  |  |
|  |  |  |  |  |
|  |  |  |  |  |
| **Dinner (6:00-7:00pm)** | **Food/drink** | **Amount** | **Household measurement** | **Remarks** |
|  |  |  |  |  |
|  |  |  |  |  |
|  |  |  |  |  |
|  |  |  |  |  |
|  |  |  |  |  |
|  |  |  |  |  |
| **Others** | **Food/drink** | **Amount** | **Household measurement** | **Remarks** |
|  |  |  |  |  |
|  |  |  |  |  |
|  |  |  |  |  |
|  |  |  |  |  |

**Section E: Nutrition Supplementation use**

| S.N. | Questions | Response | Code | Remark |
| --- | --- | --- | --- | --- |
| 1 | As an athlete, do you use supplements like protein multivitamin, caffein e.t.c | Yes | 1 |  |
|  |  | No | 0 |  |
|  | If yes, |  |  |  |
| 1.1 | Which supplement do you use? | …………… |  |  |
